# Developing and evaluating machine learning models in the prediction of recurrent sudden cardiac arrest

**DOI:** 10.3389/fcvm.2026.1883279

**Published:** 2026-10-14

**Authors:** Paulomi Gohel, Michael Daidone, Tanmay A. Gokhale, Nathan Riek, Floyd Thoma, Suresh Mulukutla, Seung J. Moon, Chinmay Patel, Sandeep K. Jain, Murat Akcakaya, Salah Al-Zaiti, Samir Saba

**Affiliations:** 1 Department of Medicine, Division of Cardiology and the Heart and Vascular Institute at the University of Pittsburgh Medical Center, Pittsburgh, PA, United States; 2 School of Engineering at the University of Pittsburgh, Pittsburgh, PA, United States; 3 School of Nursing, University of Rochester, Rochester, NY, United States

**Keywords:** machine learning, prediction models, recurrence, sudden cardiac arrest, ventricular arrhythmias

## Abstract

**Background:**

Sudden cardiac arrest (SCA) is a major killer of adults in the United States and has a significant recurrence rate. Predicting the risk of recurrent SCA has implications to patients’ counseling and management.

**Objective:**

The goal of this study is to develop and evaluate machine learning (ML) models to predict recurrent SCA in this high-risk population.

**Methods:**

Patients (*n* = 5,286) whose index arrest was coded as a primary diagnosis were selected and followed to the endpoint of second SCA. The cohort was divided into training (*n* = 3,382, 3.8% event rate), internal validation (*n* = 846, 3.8% event rate), and holdout test (1,058, 3.8% event rate) sets. External validation was performed using a cohort of 1,776 patients (2.3% event rate). Multiple ML discriminators were tested using model area under the receiver operating characteristic curve (AUROC), sensitivity, specificity, positive (PPV) and negative (NPV) predictive values, and F1 scores. The model with highest AUROC was evaluated on a holdout test set and was externally validated.

**Results:**

The gradient boosting (GB) model outperformed other models with AUROC of 0.93 in the internal validation set. It also achieved high discrimination in the holdout (AUROC=0.92), and external validation (AUROC = 0.90, sensitivity = 0.92, specificity = 0.83, F1 score = 0.19) sets. Shapley additive explanations (SHAP) demonstrated that a diagnostic code of shockable rhythm (ventricular tachycardia), evidence of structural heart disease, conduction abnormalities, and initial blood work results at the time of the index arrest were main contributors to model prediction. Low (NPV ≥ 99%), intermediate, and high (PPV≥25%) risk groups defined from the internal validation set were applied to the holdout and external validation sets with strong prediction of time to recurrent SCA (*p* = 0.003).

**Conclusion:**

Our ML prediction model demonstrates very good and stable discrimination in the prediction of recurrent SCA based on explainable clinical factors. External validation results confirm the generalizability of our findings. This model could support risk stratification and counseling for survivors of SCA.

## Introduction

Sudden cardiac arrest (SCA) represents a major public health burden, accounting for approximately 230,000 to 350,000 deaths annually in the United States and up to 50% of all cardiovascular deaths in industrialized nations (1–3) Current guidelines provide a Class I recommendation for insertion of implantable cardioverter-defibrillator (ICD) in SCA survivors with ventricular arrhythmias not due to reversible causes, based on randomized trials demonstrating 28% reduction in all-cause mortality (4, 5).

Significant gaps exist, however, in the evidence supporting secondary prevention ICD therapy. The concept of reversible cause, which would preclude ICD implantation, remains poorly defined and inconsistently applied in clinical practice, with no standardized framework for determining true reversibility (4, 6). Approximately 52% of ICD recipients never receive appropriate therapy within 10 years of implantation, and robust predictive models for recurrent SCA are lacking, with existing models achieving only modest discrimination (7–10) The goal of the present analysis was therefore to train, test and externally validate a predictive model of recurrent SCA using machine learning (ML) approaches.

## Materials and methods

### Patient population

This research was approved by the University of Pittsburgh internal review board, who waived the need for informed consent, given the retrospective, observational nature of the study. The population cohort was compiled using the University of Pittsburgh Medical Center Clinical Analytics department. Briefly, all patients (*n* = 22,369) presenting to the emergency department at any of the 40 hospitals of the institution from April 1st, 2010 to October 21st, 2024 and had a discharge diagnostic code of ventricular tachycardia (international classification of disease VT codes 427.1 and I47.2), ventricular fibrillation or flutter (VF codes 427.41, 427.42, I49.01, and I49.02), or unspecified SCA (codes 427.5, I46.2, I46.8, and I46.9) were selected. After excluding patients with prior history of VT, VF, or unspecified SCA, those with prior history of implantable cardiac defibrillator, and patients with secondary diagnoses of cardiac arrest (e.g., due to massive bleeding or sepsis), a total of 5,286 patients were retained and followed to the primary endpoint of second SCA, through May 29th, 2025. Second SCA was defined as a new hospital admission after discharge following the index admission for SCA, with discharge diagnostic code for VT, VF, or unspecified SCA, as described above. Patients admitted with a second SCA who died during the same admission were included in the event rate. Of note, discharge diagnostic codes of VT, VF, or unspecified SCA were not mutually exclusive for both the index and recurrent admissions.

The overall cohort was divided into training (*n* = 3,382, 3.8% event rate), an internal validation (*n* = 846, 3.8% event rate), and holdout test (1,058, 3.8% event rate) sets using stratified random sampling. In addition, external validation was performed using a cohort of 1,776 patients (2.3% event rate) obtained from Pinnacle Hospital in Harrisburg, Pennsylvania, which was not included in the initial analytic data pull as this hospital was only acquired by the University of Pittsburgh Medical Center in more recent years. Admission dates for the external validation cohort ranged from October 2016 to September 2025. Baseline characteristics of patients in the external validation cohort are shown in Supplementary Table S1. Five ML classifiers were trained and tested for their discrimination ability in predicting recurrent SCA (output = yes/no)5. These included logistic regression, decision tree, random forest, gradient boosting (GB), and neural network. Model hyperparameters were specified manually before model evaluation and were based primarily on the default values implemented in the corresponding scikit-learn estimators, with limited manual selection of model architecture where required, as detailed thereafter. Formal hyperparameter optimization using grid search, randomized search, or Bayesian optimization was not performed. The internal validation, holdout test, and external validation datasets were not used to select or modify model hyperparameters. Logistic regression was fitted using a maximum of 1,000 iterations with otherwise default settings (L2 regularization, LBFGS optimizer, and regularization strength C = 1.0). A decision tree classifier was trained using the default impurity criterion (Gini impurity) and default tree-growing parameters, with a fixed random seed (random state = 42). A random forest classifier consisting of 100 decision trees with a maximum tree depth of 3 was trained using bootstrap aggregation and default feature sampling (random state = 42). A GB classifier employing sequential additive decision trees with default learning rate (0.1), 100 boosting stages, and default tree depth of 3 was trained (random state = 42). Finally, a multilayer perceptron neural network with two hidden layers (32 and 16 neurons), a maximum of 200 training iterations, the default ReLU activation function, Adam optimizer, and default regularization parameters was trained (random state = 42).

Models were trained and tested on all 176 features available in the dataset, including patient demographic information, past medical history, social determinants of health, medications, electrocardiographic and echocardiographic data, as well as laboratory work, vital signs and procedures obtained at the time of the index SCA. No data were included in the models following the index SCA admission to avoid data leakage. No data were rescaled or modified in any way prior to analysis, except for imputation of missing data, as described below. For each ML model, we calculated model accuracy, area under the receiver operating characteristic curve (AUROC), sensitivity, specificity, positive (PPV) and negative (NPV) predictive values, and F1 scores. The model with highest AUROC was chosen for evaluation on the holdout test set and for external validation.

Model performance was further tested by defining risk groups based on the internal validation test set and further applying the same cutoffs to the holdout test and external validation sets. Predicted probabilities from the GB model were calibrated to improve agreement between predicted and observed event rates, using isotonic regression. The calibration model was fit using the training dataset with 5-fold cross-validation to prevent overfitting (results shown in Supplementary Table S2 in the Supplemental Materials). The learned calibration map was then applied to generate calibrated predicted probabilities for the holdout test and the external validation datasets. Calibration plots with slope, intercept and Brier scores are shown in Supplementary Figure S1. Risk score cutoffs were applied to the calibrated probabilities of the internal validation set to define a low-risk group for recurrent SCA based on a NPV ≥ 99% and a high-risk group based on a PPV ≥ 25%. Patients were classified in an intermediate risk group if they did not meet either cutoff criterion. Lastly, Shapley additive explanations (SHAP) analyses were performed on the holdout and external validation test sets to provide model explainability and generalizability, based on clinical data that contributed the most to model performance.

### Data collection and imputation

For each patient in the cohort, data were collected from the electronic health records preceding and concurrent with the index SCA admission. Data collected included demographic information (e.g., age, sex, race, ethnicity), vital measurements (e.g., height, body mass index), vital signs (e.g., heart rate, blood pressure), laboratory data (e.g., serum magnesium), known comorbidities (e.g., hypertension), home medications with primary focus on cardiovascular medications (e.g., beta-blockers, angiotensin converting enzyme inhibitors), prior electrocardiographic and echocardiographic data. Variables with >20% missing data were excluded from analyses. Missing data were otherwise imputed to the mean of continuous variables and to the mode of categorical variables. Sensitivity analysis performed were performed demonstrating that study findings are independent of imputation method. These data are shown in Supplementary Table S3 of the Supplementary Materials.

### Statistical analysis

Continuous data are presented as mean ± standard deviation and are compared using the *t* test between patients who suffered a recurrent SCA versus those who did not. Categorical data are reported as frequencies with percentages and are compared using Chi-square test. Five classifiers (Logistic Regression, Decision Tree, Random Forest, Gradient Boosting and Neural Network) were evaluated with performance assessed using accuracy, AUROC, sensitivity, specificity, PPV, NPV, and the F1 score, which is the harmonic mean of precision (PPV) and recall (sensitivity) (11)(F1 = 2 × sensitivity × PPV/ (sensitivity + PPV)). These parameters were reported at the threshold that maximizes Youden's index, which is equal to (sensitivity + specificity – 1) (12, 13). The Youden threshold was used solely to provide a standardized data-derived operating point at which threshold-dependent measures, including sensitivity and specificity, could be summarized across models. It was not intended to define the thresholds for clinical risk stratification, which are defined above. Kaplan Meier survival curves free from recurrent SCA were constructed for the holdout and external validation test sets at the same cutoffs derived from the calibrated results of the internal validation dataset, as noted above. Statistical significance was tested at the 0.05 cutoff for *p* values. All analyses were performed using open-source Python coding (version 3.11.5, Python Software Foundation, Wilmington, Delaware) with scikit-learn (version 2.1.3).

## Results

### Patient population

Baseline characteristics of our derivation, validation and testing cohort, stratified by recurrence of SCA are shown in Table 1. Of the overall cohort of 5,286 who experience an initial SCA, 202 patients suffered a recurrent arrest at a median follow-up time of 197 days (Figure 1). As noted, patients with recurrent SCA were more likely to be men, to have a diagnosis of coronary artery disease and congestive heart failure. They were also more likely to have a diagnosis of shockable rhythm (VT or VF) at the time of the index SCA. Electrocardiographically, patients with recurrent arrest had significantly wider QRS and QT intervals.

**Table 1:** Characteristics of patients in the training, validation, and testing datasets stratified by recurrent SCA status at the time of presentation to the emergency department with index arrest.

Variable	Total	No Recurrent SCA	Recurrent SCA	*P* value
Number	5,286	5,084	202	
Age	67 ± 16	68 ± 16	66 ± 14	0.15
Women	2,185 (41%)	2,135 (42%)	50 (25%)	<0.001
Race				0.36
White	4,293 (81%)	4,125 (81%)	168 (83%)	
Black	900 (17%)	867 (17%)	33 (16%)	
Other	93 (2%)	92 (2%)	1 (1%)	
Ethnicity				0.38
Non-Hispanic	4,633 (88%)	4,458 (88%)	175 (87%)	
Hispanic	35 (1%)	35 (1%)	0 (0%)	
Not specified	618 (12%)	591 (12%)	27 (13%)	
Hypertension	3,982 (75%)	3,849 (76%)	133 (66%)	0.002
Diabetes mellitus	1,348 (25%)	1,292 (25%)	56 (28%)	0.46
Atrial fibrillation	871 (16%)	833 (16%)	38 (19%)	0.38
Coronary artery disease	1,388 (26%)	1,295 (25%)	93 (46%)	<0.001
Congestive heart failure	1,156 (22%)	1,074 (21%)	82 (41%)	<0.001
Obstructive lung disease	764 (14%)	739 (14%)	25 (12%)	0.47
Chronic kidney disease	666 (13%)	646 (13%)	20 (10%)	0.28
Charlson comorbidity index	2.7 ± 1.9	2.7 ± 1.9	2.5 ± 1.9	0.14
Index SCA				<0.001
Ventricular fibrillation	640 (12%)	561 (11%)	79 (39%)	
Ventricular tachycardia	812 (15%)	646 (13%)	166 (82%)	
Unspecified SCA	4,644 (88%)	4,588 (90%)	45 (22%)	
Left ventricular ejection fraction	42 ± 6	42 ± 6	41 ± 10	0.26
Heart rate (beat per minute)	95 ± 23	96 ± 22	94 ± 35	0.59
QRS duration (millisecond)	106 ± 31	105 ± 31	124 ± 35	<0.001
QT interval (millisecond)	402 ± 63	401 ± 62	425 ± 70	<0.001
Estimated household income ($K)	48 ± 17	48 ± 17	50 ± 18	0.12
Beta blockers	3,142 (59%)	3,016 (59%)	76 (38%)	0.42
ACEi/ARB/ARNI	2,062 (39%)	1,951 (38%)	111 (55%)	0.037

ACEi, angiotensin converting enzyme inhibitor; ARB, angiotensin receptor blocker; ARNI, angiotensin receptor neprilysin inhibitor; SCA, sudden cardiac arrest.

**Figure 1:**
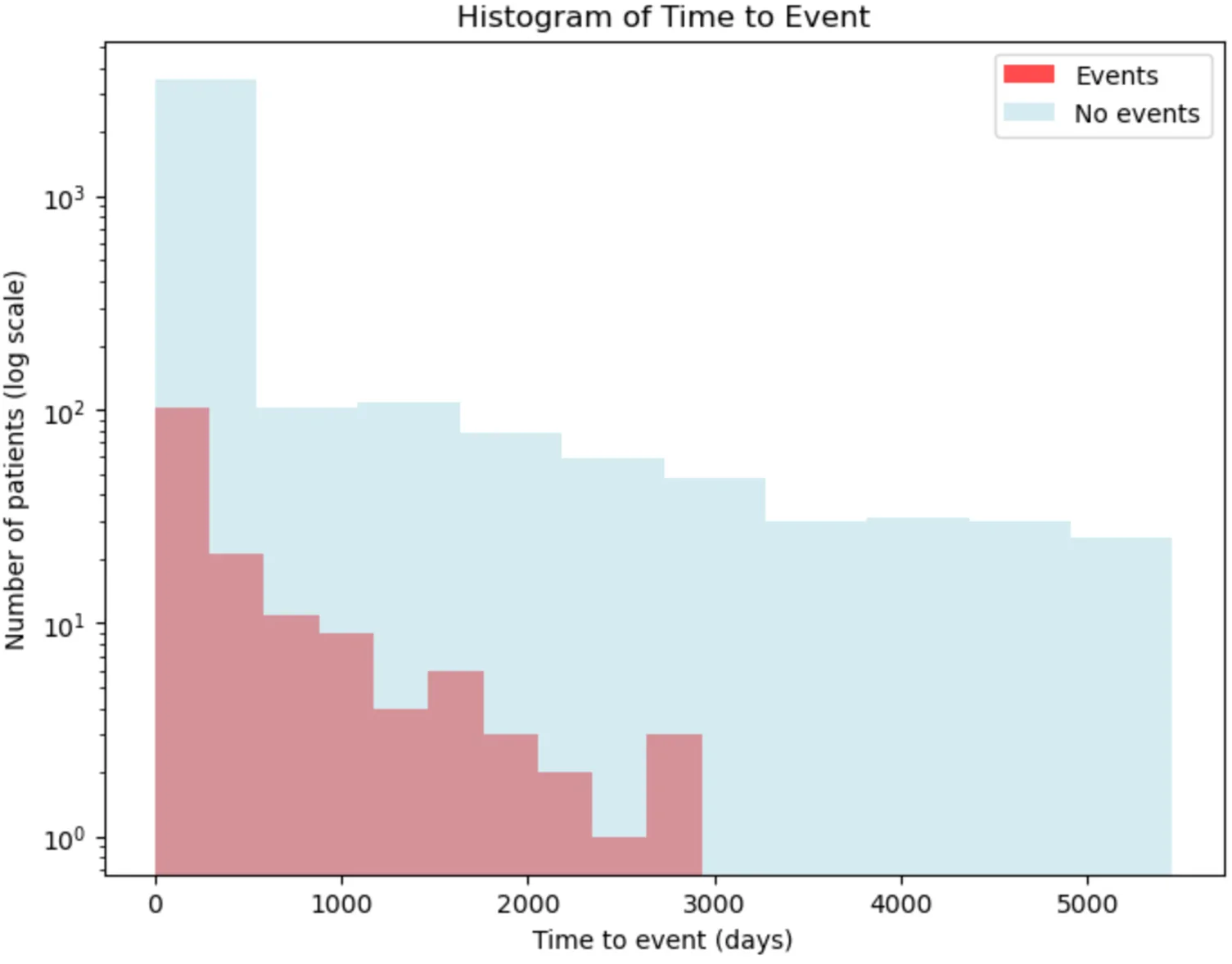
Histogram of time to event in patients with (red bars) versus without (blue bars) recurrent SCA.

### ML model performance

Multiple ML models were trained and tested on the internal validation dataset. The strongest performing model was selected based on higher levels of discrimination, as measured by AUROC. As shown in Table 2 and Figure 2, the GB model was the highest performing. All further analyses were performed with the GB classifier. Across the 5 held-out folds of the training cohort, the mean AUROC was 0.92 ± 0.02, mean PRAUC was 0.32 ± 0.06, and mean Brier score was 0.033 ± 0.002 (Table 2 and Supplementary Table S2). These results were consistent with performance in the internal validation cohort (AUROC, 0.935; PRAUC, 0.255; Brier score, 0.033) and the holdout test cohort (AUROC, 0.922; PRAUC, 0.252; Brier score, 0.034). Discrimination remained high in the external validation cohort, with an AUROC of 0.903, although PRAUC was lower at 0.164 in the setting of lower external event prevalence (Table 2).

**Table 2:** Performance of candidate ML models in the internal validation cohort (upper section) and performance of selected gradient boosting classifier across the cross-validated training, internal validation, hold out test, and external validation cohorts (lower section).

Performance of candidate ml models in the internal validation cohort									
Model	Accuracy	AUROC	Sens	Spec	PPV	NPV	F1 Score	PRAUC	Brier Score
Logistic Regression	0.843	0.893	0.871	0.842	0.173	0.994	0.289	0.314	0.033
Decision Tree	0.924	0.604	0.258	0.950	0.163	0.971	0.200	0.065	0.071
Random Forest	0.820	0.929	1.000	0.813	0.169	1.000	0.290	0.313	0.030
Gradient Boosting	**0**.**878**	**0**.**935**	**1**.**000**	**0**.**874**	**0**.**231**	**1**.**000**	**0**.**376**	**0**.**255**	**0**.**033**
Neural Network	0.826	0.867	0.806	0.827	0.151	0.991	0.254	0.195	0.045

Testing of selected gradient boosting model across development and validation cohorts									
Dataset	Accuracy	AUROC	Sens	Spec	PPV	NPV	F1 Score	PRAUC	Brier Score
Training Cohort (mean of 5-fold cross validation)^a^	0.989	0.920	0.969	0.990	0.789	0.999	0.870	0.324	0.033
Internal Validation^b^	0.878	0.935	1.000	0.874	0.231	1.000	0.376	0.255	0.033
Holdout Test	0.871	0.922	0.925	0.868	0.216	0.997	0.351	0.252	0.034
External Validation	0.833	0.903	0.854	0.832	0.107	0.996	0.191	0.164	0.026

Bold row indicates the best performing model.

AUROC, Area under the receiver operating characteristic curve; Sens, sensitivity; Spec, specificity; PPV, Positive predictive value; NPV, Negative predictive value; F1 score, 2×(PPV × sensitivity)/(PPV + sensitivity); PRAUC, area under the precision recall curve.

a Results for the training cohort represent mean performance across the held-out portions of 5 stratified cross-validation folds. All other results were obtained by applying the final fitted model to the indicated dataset. The internal validation, holdout test, and external validation datasets were not used to fit the model or select its hyperparameters.

b The internal validation cohort was used to compare candidate model classes, whereas the holdout test and external validation cohorts were reserved for evaluation of the selected model.

**Figure 2:**
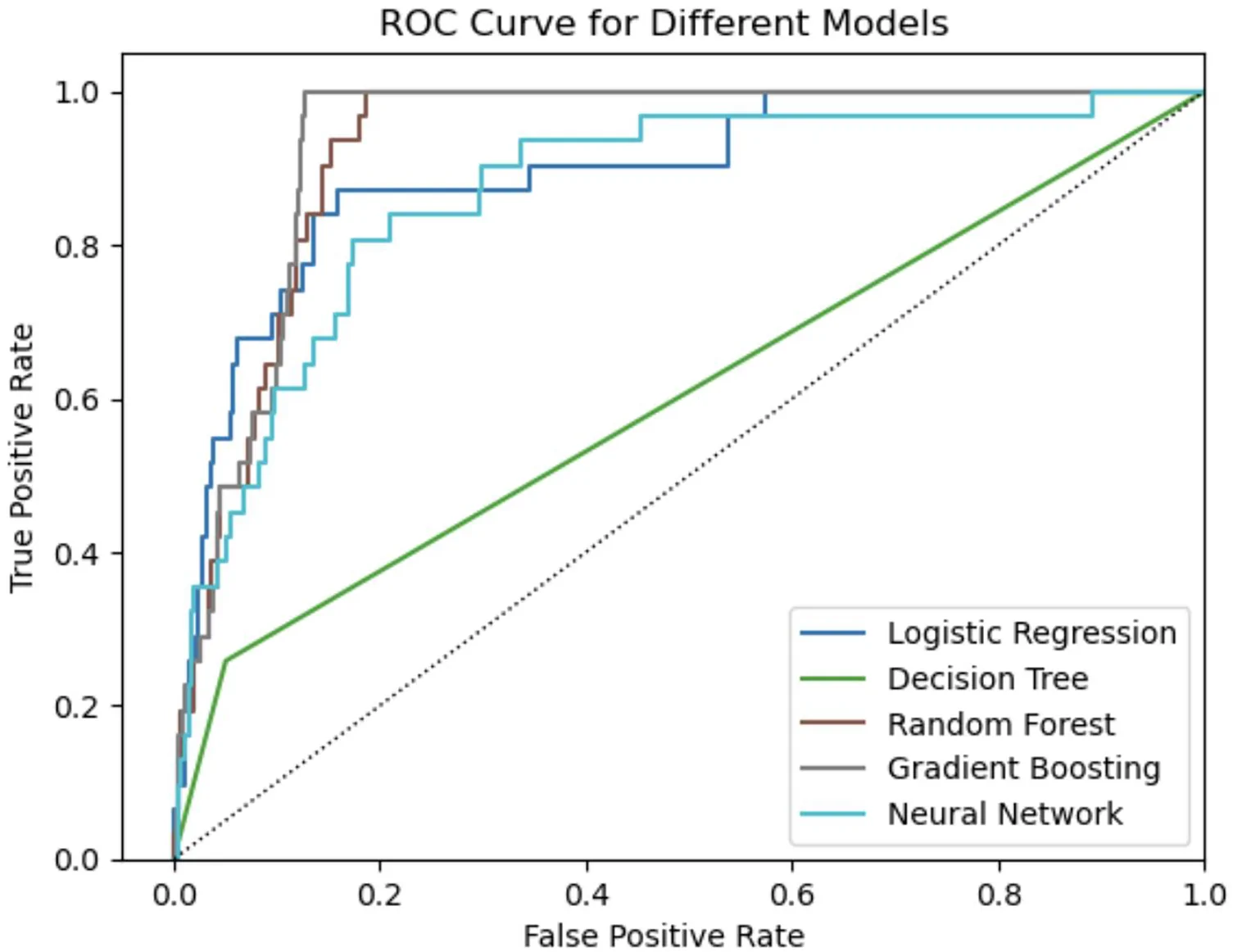
Area under the receiver operating characteristic (ROC) curve for several machine learning models applied to the internal validation dataset. Note that the Gradient Boosting and Random Forest models had the best area under the ROC curve.

Applying the GB model to the holdout set demonstrated strong discrimination with an accuracy of 0.87, AUROC of 0.92, sensitivity of 0.92, specificity of 0.87, and F1 score of 0.35 (Table 2, and Figure 3A). The same model was applied to the external validation dataset, where model performance was maintained with an accuracy of 0.83, AUROC of 0.90, sensitivity of 0.85, specificity of 0.83 (Table 2, and Figure 3B). The F1 score dropped from 0.35 to 0.19, reflecting the lower event rate in the external validation set (2.3%) compared to the holdout test set (3.8%). It is worth noting that median time to second SCA was shorter (169 days) in the external validation dataset compared to the training and testing datasets (197 days).

**Figure 3:**
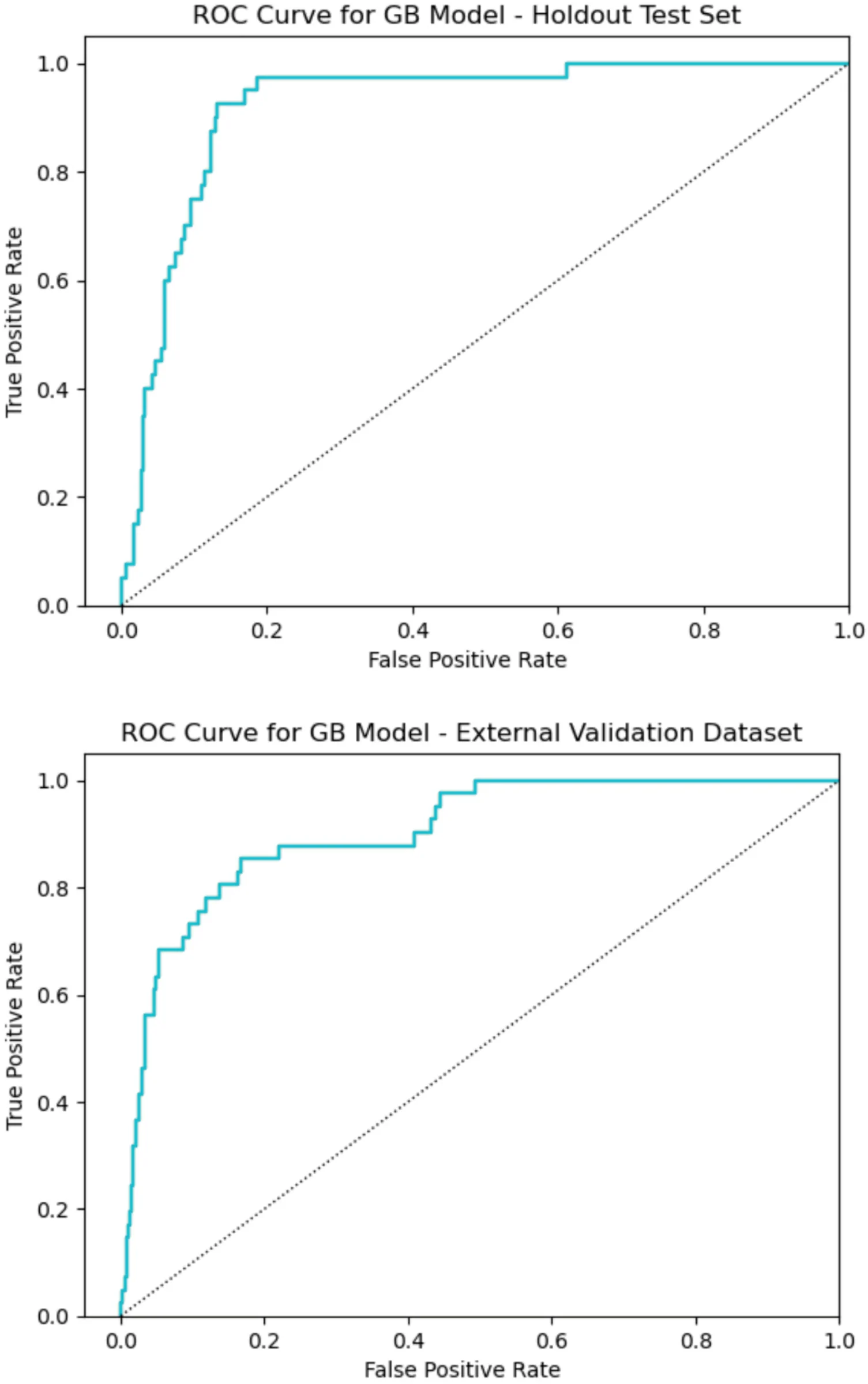
Receiver operating characteristic (ROC) curve of the gradient boosting model applied to the holdout test set (upper panel) and to the external validation dataset (lower panel).

SHAP analyses were applied to the holdout test set (Figure 4**, upper panel**) and to the external validation set (Figure 4**, lower panel**) to provide model explainability. It is worth noting that the top 20 clinical features contributing to model performance are virtually the same for both datasets, supporting the generalizability of the model. It can be noted that the key predictors of recurrent SCA are the presence of a diagnostic code for shockable rhythm (e,g., VT) at the time of the index arrest and the insertion of an implantable cardioverter defibrillator during that index hospital admission. Other important parameters contributing to model performance include serum blood work and vital signs at the time of the original SCA, as well as echocardiographic (e.g., ejection fraction) and electrocardiographic (e.g., QRS width) data. Lastly, testing and interventions (e.g., defibrillator implantation) at the time of the index SCA also play a role in predicting future recurrent events.

**Figure 4:**
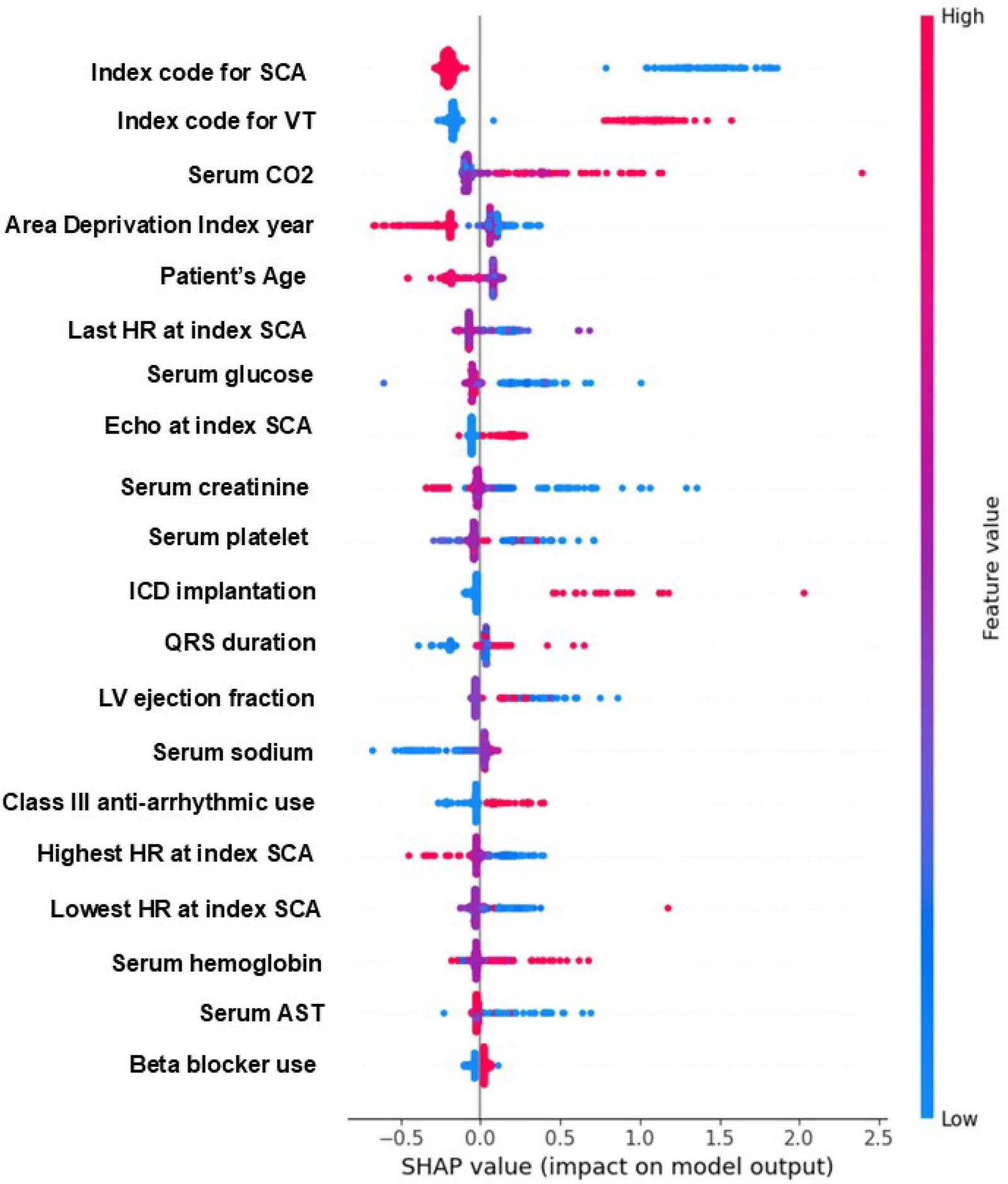

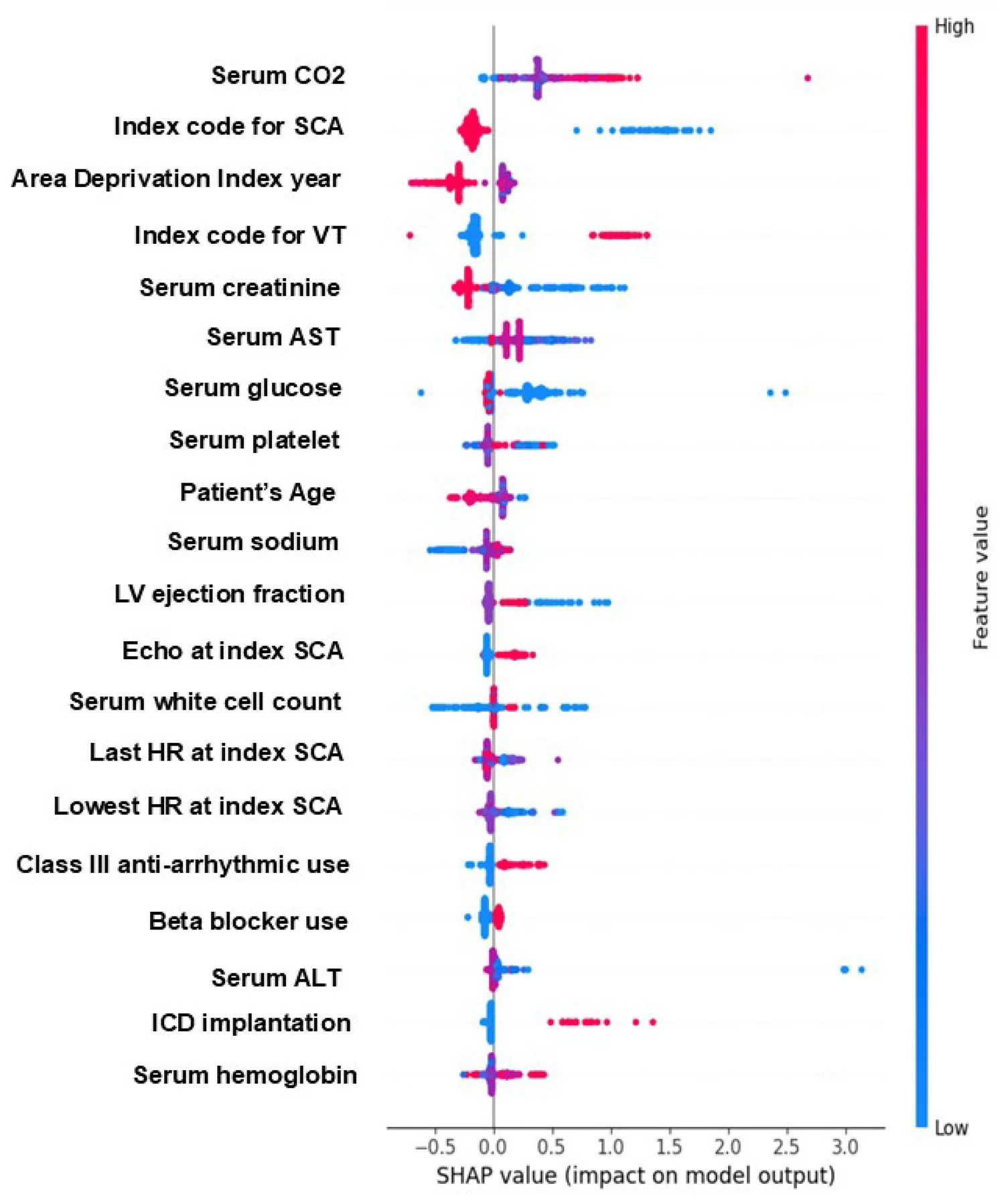
Shapley additive explanations analysis applied to the holdout test set (upper panel) and to the external validation set (lower panel) demonstrating the top 20 parameters contributing to the prediction model.

Patients in the holdout test set and the external validation dataset were further divided into low, intermediate, and high- risk groups based on cutoff values applied to the calibrated risk probabilities in the internal validation dataset as generated by the GB model. As seen in Figure 5, the time to second SCA was significantly shorter in higher predicted risk groups (*p* = 0.003), further demonstrating the robust performance of the GB model in predicting recurrent SCA events.

**Figure 5:**
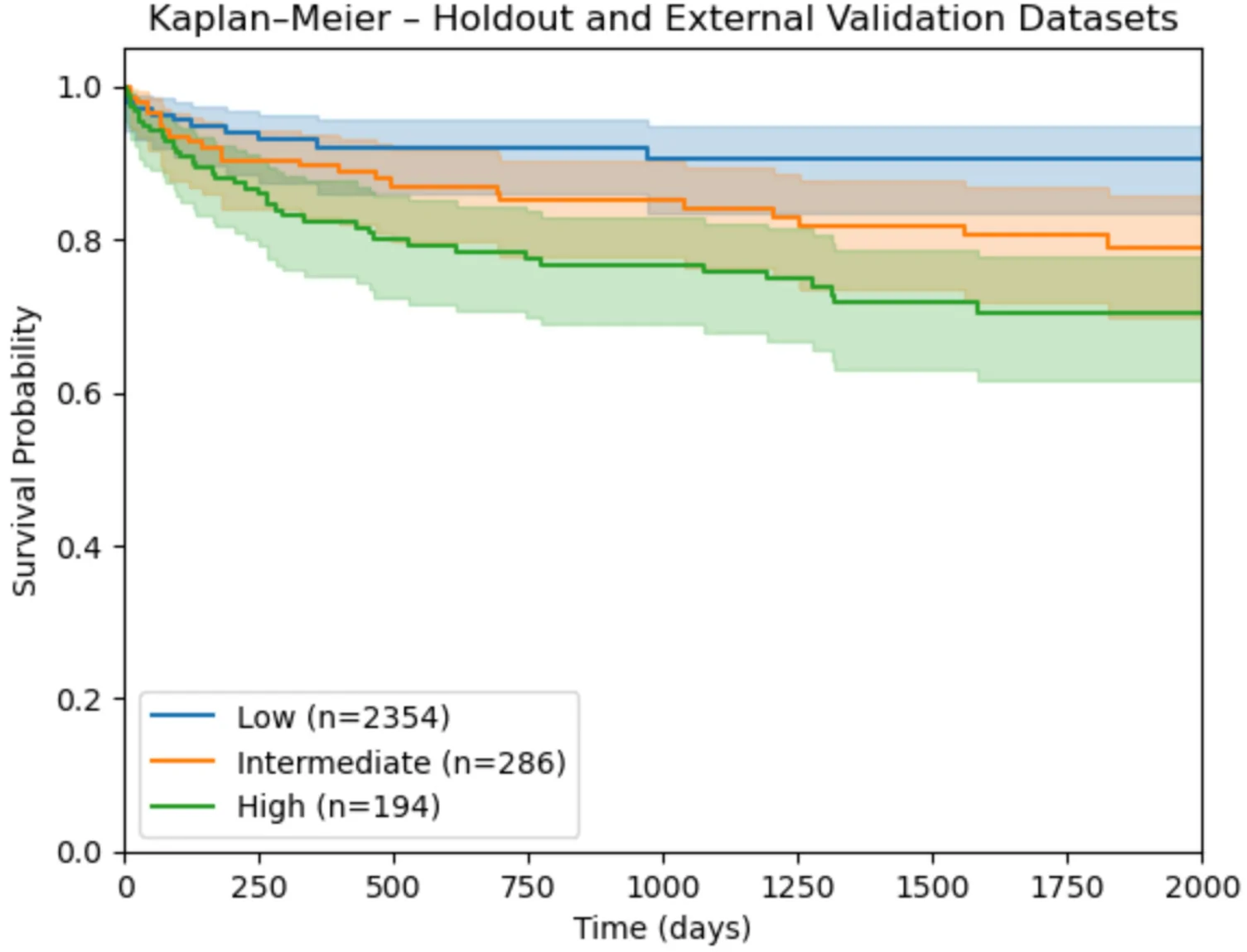
Kaplan meier survival free from recurrent sudden cardiac arrest stratified by risk group in the combined holdout test and external validation datasets. The high risk (PPV > 25%) and low risk (NPV > 99%) groups are defined based on cutoffs applied to the risk score of the training internal validation dataset (log-rank *p* value = 0.003).

## Discussion

In this study, we developed and externally validated a ML model to predict recurrence of SCA in patients who survived an initial event. Among the candidate algorithms evaluated, the GB model demonstrated superior predictive performance and achieved excellent discrimination in the external validation cohort, with an AUROC approaching 0.90, indicating a high discrimination capacity to identify patients at elevated risk for recurrent SCA. The robustness of the model across independent datasets supports its potential generalizability and suggests that ML approaches can meaningfully enhance risk stratification in this population. Given the substantial morbidity and mortality associated with recurrent malignant ventricular arrhythmias following an initial SCA, improved prediction tools may enhance individualized patient counseling and inform management strategies in survivors of SCA.

A key strength of the present model is its interpretability through SHAP analysis, which allows quantification of both the relative importance and directionality of predictors contributing to model output (14). Our findings demonstrate consistency between SHAP variables derived from the internal holdout and the external validation sets, further establishing the generalizability of our prediction model. Several of the highest-ranking variables identified by the model are biologically plausible and consistent with established mechanisms underlying malignant ventricular arrhythmias. For example, the presence of VT was among the stronger predictors of recurrent SCA, with higher values associated with increased risk. This finding aligns with the well-recognized role of sustained VT as a primary, scar-mediated, non-reversible mechanism responsible for sudden cardiac death (15, 16). Similarly, electrocardiographic markers such as prolonged QRS duration were associated with increased predicted risk, likely reflecting underlying myocardial scar, conduction abnormalities, or structural heart disease that predispose to ventricular arrhythmogenesis. Measures of myocardial function demonstrated the expected relationships, with higher left ventricular ejection fraction being associated with lower predicted risk of recurrent SCA. Importantly, the SHAP framework also allows visualization of the directionality of individual predictors, providing clinically interpretable insight into how specific physiological or clinical characteristics influence predicted risk. The ability to combine strong predictive performance with transparent interpretability represents a key advantage of contemporary ML models applied to cardiovascular risk prediction (17).

Risk stratification after survival from a prior SCA remains an important clinical challenge. Current clinical decision-making regarding secondary prevention primarily focuses on identifying reversible causes of SCA. According to contemporary guidelines, insertion of an ICD is recommended for survivors of VT or VF causing hemodynamic instability or cardiac arrest when the event is not attributable to a reversible cause (16, 18). While these recommendations have substantially improved outcomes in patients at high risk of recurrent arrhythmias, determining the likelihood of recurrence in individual patients remains complex. Traditional risk stratification approaches have largely relied on a limited number of clinical variables, such as a reduced left ventricular myocardial function, despite the recognition that many sudden cardiac events occur in individuals who do not meet conventional risk thresholds (15, 19). Prior attempts at predicting recurrent SCA have identified traditional cardiac risk factors such as the presence of coronary disease, hypertension or diabetes mellitus (20), or have used ML approaches but with moderate discrimination ability (AUROC = 0.73) (10). In the present context, ML models capable of integrating large numbers of clinical variables and capturing nonlinear relationships may offer important advantages. The strong predictive performance observed in our study suggests that such approaches may complement existing clinical assessment and potentially refine risk stratification among survivors of SCA.

Prior studies attempting to improve prediction of SCA or malignant ventricular arrhythmias have generally demonstrated modest predictive performance. Multivariable models based on conventional statistical approaches often report discrimination in the range of approximately 0.65 to 0.80 (15, 21). In contrast, GB methods can identify complex interactions among predictors that may not be captured by traditional regression models. The present GB model achieved substantially higher discrimination in external validation, suggesting that ML approaches may provide incremental value in identifying patients at risk for recurrent SCA. Importantly, the integration of SHAP-based explainability addresses a common criticism of ML in clinical medicine, namely the perception that these methods function as ‘black boxes’, by providing clear insight into the clinical variables driving model prediction.

Several limitations of this study should be considered. First, the overall number of recurrent SCA events in the dataset was relatively small, which may limit statistical power and increase susceptibility to model overfitting despite the use of validation techniques. This however is an inherent problem in SCA, which is the ultimate example of rare but very high-consequence clinical event. Second, the analysis was based on retrospective, observational data and is therefore subject to potential inaccuracies in coding, missing data, unmeasured confounding, and ascertainment bias. We have, however, mitigated this limitation by excluding any variable with >20% missing values. Third, although the model demonstrated strong performance in an external validation cohort, further validation in additional populations and across different healthcare systems will be necessary to establish generalizability. Fourth, model hyperparameters were selected manually and were based predominantly on commonly used default values rather than systematic hyperparameter optimization. Although this approach limits conclusions regarding whether performance could be improved through further tuning, it also reduced model-selection complexity, and performance was subsequently evaluated in separate internal validation, holdout test, and external validation cohorts. Finally, the model was limited to variables available within the dataset and did not incorporate potentially informative predictors such as advanced cardiac imaging parameters, electrophysiologic testing, or genetic data, which may further refine risk prediction, but limit its scalability.

In conclusion, we developed and externally validated a GB model capable of accurately predicting the risk of recurrent SCA following an initial event. The model demonstrated excellent discrimination and identified clinically meaningful predictors through SHAP-based explainability analysis. With further validation, such predictive tools may help refine individualized risk stratification and support clinical decision-making in survivors of SCA, potentially complementing current guideline-directed strategies for secondary prevention.

## Data Availability

The raw data supporting the conclusions of this article will be made available by the authors, without undue reservation.
