# Supplementary material for: Developing and evaluating machine learning models in the prediction of recurrent sudden cardiac arrest
Source: Front Cardiovasc Med. 2026 Oct 14;13:1883279. doi: 10.3389/fcvm.2026.1883279 (PMC13713392; doi:10.3389/fcvm.2026.1883279)
Supplement: Supplementary file 1 [file Table1.docx]

**Supplemental Materials**

**Table S1.** Baseline characteristics of patients in the external validation cohort

|  | **Total** | **No Recurrent SCA** | **Recurrent SCA** | **P value** |
| --- | --- | --- | --- | --- |
| **Number** | 1,776 | 1,735 | 41 |  |
| **Age** | 67±16 | 67±16 | 66±12 | 0.75 |
| **Women** | 618 (35%) | 605 (35%) | 13 (33%) | 0.74 |
| **Race**  *White*  *Black*  *Other* | 1,428 (80%)  276 (16%)  72 (4%) | 1,391 (80%)  272 (16%)  72 (4%) | 37 (90%)  4 (10%)  0 (0%) | 0.21 |
| **Ethnicity**  *Non-Hispanic*  *Hispanic*  *Not specified* | 1,689 (95%)  57 (3%)  30 (2%) | 1,650 (95%)  56 (3%)  29 (2%) | 39 (95%)  1 (2%)  1 (3%) | 0.90 |
| **Hypertension** | 1,537 (86%) | 1,504 (87%) | 36 (80%) | 0.25 |
| **Diabetes mellitus** | 383 (22%) | 363 (21%) | 20 (49%) | <0.001 |
| **Atrial fibrillation** | 232 (13%) | 212 (12%) | 20 (49%) | <0.001 |
| **Coronary artery disease** | 362 (20%) | 335 (19%) | 27 (66%) | <0.001 |
| **Congestive heart failure** | 411 (23%) | 381 (22%) | 30 (73%) | <0.001 |
| **Obstructive lung disease** | 180 (10%) | 173 (10%) | 7 (17%) | 0.18 |
| **Chronic kidney disease** | 246 (14%) | 235 (13%) | 11 (27%) | 0.022 |
| **Charlson comorbidity index** | 2.8±1.7 | 2.8±1.7 | 3.6±2.4 | <0.001 |
| *Index SCA*  *Ventricular fibrillation*  *Ventricular tachycardia*  *Unspecified SCA* | 348 (20%)  150 (8%)  139 (8%) | 312 (18%)  26 (7%)  108 (6%) | 36 (88%)  24 (58%)  31 (76%) | <0.001 |
| **Left ventricular ejection fraction** | 46±7 | 46±6 | 44±9 | 0.092 |
| **Heart rate (beat per minute)** | 102±29 | 103±29 | 87±21 | <0.001 |
| **QRS duration (millisecond)** | 92±22 | 92±22 | 99±30 | 0.16 |
| **QT interval (millisecond)** | 390±39 | 389±38 | 412±57 | 0.018 |
| **Estimated household income ($K)** | 58±17 | 58±13 | 59±14 | 0.78 |
| **Beta blockers** | 707 (40%) | 635 (39%) | 72 (78%) | <0.001 |
| **ACEi/ARB/ARNI** | 583 (33%) | 561 (32%) | 22 (54%) | <0.001 |

**Table S2.** Five-fold cross validated performance of the gradient boosting model in the development training cohort

| **Fold** | **N** | **Event Rate (%)** | **AUROC** | **PRAUC** | **Brier Score** |
| --- | --- | --- | --- | --- | --- |
| **1** | 677 | 3.84 | 0.94 | 0.36 | 0.031 |
| **2** | 677 | 3.99 | 0.89 | 0.37 | 0.031 |
| **3** | 676 | 3.85 | 0.91 | 0.23 | 0.036 |
| **4** | 676 | 3.85 | 0.92 | 0.35 | 0.033 |
| **5** | 676 | 3.85 | 0.94 | 0.31 | 0.033 |
| **Mean ± SD** | -- | 3.87±0.06 | 0.92±0.02 | 0.32±0.06 | 0.033±0.002 |

**Table S3.** Model performance using *Median* versus *Mean* imputation methods for missing values

| **Imputation by *MEAN* and Mode** | | | | | | | | | |
| --- | --- | --- | --- | --- | --- | --- | --- | --- | --- |
| **DATASET** | **Accuracy** | **AUROC** | **Sens** | **Spec** | **PPV** | **NPV** | **F1 Score** | **PRAUC** | **Brier score** |
| **Training** | 0.989 | 0.998 | 0.969 | 0.990 | 0.789 | 0.999 | 0.870 | 0.971 | 0.009 |
| **Validation** | 0.878 | 0.935 | 1.000 | 0.874 | 0.231 | 1.000 | 0.376 | 0.255 | 0.033 |
| **Holdout Test** | 0.871 | 0.922 | 0.925 | 0.868 | 0.216 | 0.997 | 0.351 | 0.252 | 0.034 |
| **External Validation** | 0.833 | 0.903 | 0.854 | 0.832 | 0.107 | 0.996 | 0.191 | 0.164 | 0.026 |
| **Imputation by *MEDIAN* and Mode** | | | | | | | | | |
| **DATASET** | **Accuracy** | **AUROC** | **Sens** | **Spec** | **PPV** | **NPV** | **F1 Score** | **PRAUC** | **Brier score** |
| **Training** | 0.973 | 0.998 | 0.985 | 0.972 | 0.591 | 0.999 | 0.739 | 0.970 | 0.011 |
| **Validation** | 0.883 | 0.917 | 0.926 | 0.882 | 0.202 | 0.997 | 0.331 | 0.186 | 0.033 |
| **Holdout Test** | 0.949 | 0.989 | 0.975 | 0.948 | 0.419 | 0.999 | 0.586 | 0.830 | 0.015 |
| **External Validation** | 0.822 | 0.902 | 0.878 | 0.821 | 0.104 | 0.997 | 0.186 | 0.213 | 0.023 |

**Figure S1.** Calibration curves with event histogram for the Holdout test set (**panel A**) and the External Validation set (**panel B**)
